# Calcification of the alar ligament of the cervical spine in a patient with rheumatoid arthritis

**Published:** 2012-10-29

**Authors:** Rahma Boussaadani Soubai, Latifa Tahiri, Fatima Zahra Abourazzak, Siham Tizniti, Taoufik Harzy

**Affiliations:** 1 Rheumatology department, CHU Hassan II Fes, Morocco; 2 Radiology department, CHU Hassan II Fes, Morocco

**Keywords:** Alar ligament, cervical spine, calcification

## Abstract

Calcification of the alar ligament is rare. It usually develops as a result of traumatic injury and is especially prominent in the elderly. CT scanning is the gold standard of the diagnosis. We report a case of a calcification of the transverse and alar ligament in a patient with rheumatoid arthritis.

## Introduction

Calcification in the alar ligament is rare. It usually develops as a result of traumatic injury especially in the elderly. We present a case of a calcification of the transverse and alar ligament in a patient with rheumatoid arthritis.

## Patient and observation

**Figure 1:**
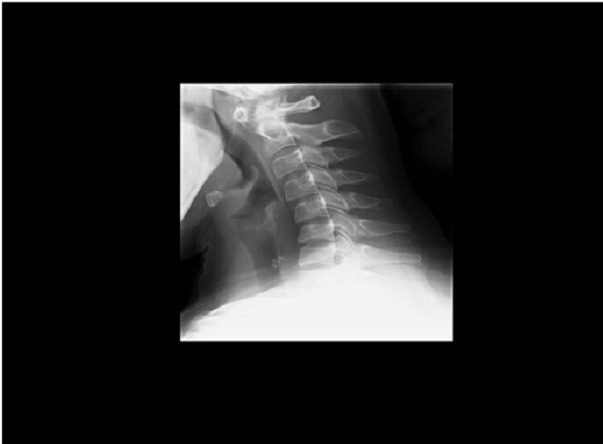
A lateral view of cervical spine radiograph showed an atloïdo-axoidien diastasis of 04 mm

**Figure 2:**
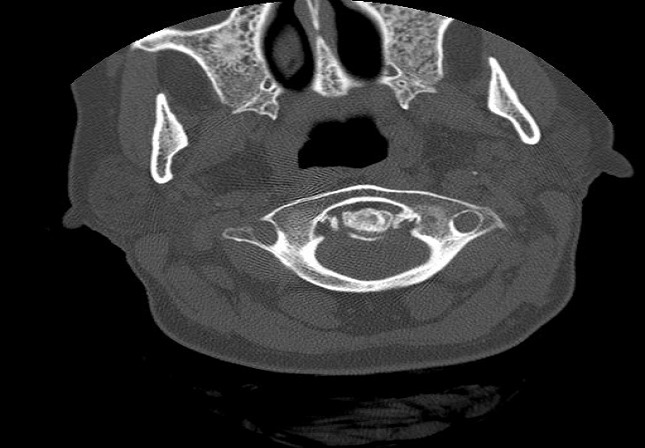
Axial computed tomography (CT) of the cervical spine revealing calcifications of transverse and alar ligaments, surrounding the odontoid process

A 34-year-old woman, without history of trauma, followed for 09 years for a rheumatoid arthritis. The patient presented a chronic nuchal pain. Physical examination found a painful palpation of C1 and C2, without stiff neck and neurologic examination was normal. A lateral view of cervical spine radiograph showed an atloïdo-axoidien diastasis of 04 mm (Figure 1). An atloïdo-axoidien dislocation was suspected, so a computed tomography (CT) of the cervical spine was carried out and revealed calcifications of transverse and alar ligaments, surrounding the odontoid process (Figure 2). The spinal canal appeared normal and no dislocation C1-C2 was detected. The patient used a cervical splint with an anti-inflammatory drug and the nuchal pain decreased gradually.

## Discussion

The alar ligaments are strong rounded structures, which arise on both sides of the upper part of the odontoid process and, passing obliquely upward and laterally, are inserted into the medial surface of the condyles of the occipital bone. They play an important role in stabilizing the head during rotatory movements at the craniovertebal junction. The transverse ligament of the atlas runs along the dorsal aspect of the odontoid process to the lateral mass of the atlas bilaterally.

In 1982, Ziza et al [1]. reported calcified deposits similar to those in the cases presented here, but they did not identify the ligament in which deposition of calcification was shown. Bouvet et al [2] described four cases in which calcified deposits in the cruciform ligament of the atlas which resolved with administration of antiinflammatory drug, and Yasukawa et al [3] and Yoshida et al [4] reported one case each in which acute nuchal pain was associated with calcified deposits in the yellow ligament of the lower cervical spine. In our patient, calcification was observed in the alar and the transverse ligaments.

Calcification in the alar ligament is very rare, Kobayashi et al [5] reported 2 cases of alar ligament calcification, and another case was reported by Sim et al [6]. It usually develops with increasing prevalence after the age of 40 years, especially in the elderly, following minor trauma [3, 4, 7]. However our report was only 34 years old, and without known injury. Other authors have reported cases of calcification of the upper cervical ligament with neurologic involvement [4, 8, 9]. No neurologic symptoms were present in our patient. CT scanning focusing on C1/C2 is the gold standard of the diagnosis. This makes it possible to identify the calcification in the upper cervical spine.

The composition of the deposits in our cases is unknown, although the components of calcification of the cervical spine in general include calcium pyrophosphate dihydrate (CPPD) and hydroxyapatite (HAP), alone or in combination [2, 3, 4, 7].

The association between rheumatoid arthritis and periodontoid calcification is not easy to interpret in that no causal relationship has yet been identified. It is most likely a chance association.

Treatment of ligament calcification of the cervical spine is controversial. When neurologic symptoms are present, surgery is usually performed. However, conservative treatment often is efficacious when neurologic symptoms are absent [2, 10]. A trial of anti-inflammatory therapy, analgesia, bed rest, and cervical splinting should be attempted prior to surgical intervention in such cases [5].

## Conclusion

Calcification in the alar ligament is rare, though few cases with calcification of the transverse or alar ligament have been reported. In some cases, it is associated with neck pain, relieved by anti-inflammatory drugs and neck immobilization. The association between rheumatoid arthritis and periodontoid is most likely a chance association.
